# Risk factors for pneumonia in children with *Bordetella pertussis* infection and co-infection status in Ningbo, China

**DOI:** 10.1515/med-2026-1425

**Published:** 2026-04-30

**Authors:** Chunyan Liu, Qian Xu, Zhuoling Li, Xiaoli Yang, Lili Guo, Xin Liu, Wenyuan Liu

**Affiliations:** Department of Clinical Laboratory, The Affiliated Women and Children’s Hospital of Ningbo University, Ningbo, Zhejiang, China; Department of Outpatient and Emergency, The Affiliated Women and Children’s Hospital of Ningbo University, Ningbo, Zhejiang, China; Department of Stomatology, The Affiliated Women and Children’s Hospital of Ningbo University, Ningbo, China

**Keywords:** risk factor, *Bordetella pertussis*, pneumonia, coinfection, children

## Abstract

**Objectives:**

Pertussis, also known as whooping cough,is a significant contributor to pneumonia cases in children. The existing literature regarding pertussis in China is sparse, highlighting the need for further research in this area. This research aimed to find out the factors related to pneumonia in B. Pertussis co-infected children.

**Methods:**

From January to April 2024,children identified with B. Pertussis infection were enrolled in the study. Subsequently, they were divided into two groups:one consisting of those without pneumonia and another comprising those with pneumonia. This study was a retrospective observational study. In this study, variables assessed included demographics, clinical symptoms and laboratory findings. The study examined the various risk factors associated with pneumonia resulting from infection with B. Pertussis in both groups.

**Results:**

Seventy-five patients participated in the study, Among the participants, individuals (77.30 %) had completed the full course of the pertussis vaccine, while 17 individuals (22.70 %) had not,with 29 belonging to the pneumonia group and 46 to the non-pneumonia group. There was borderline significance between the completion of vaccination and the occurrence of pneumonia (p=0.05). Those in the pneumonia group exhibited the highest recorded body temperature due to fever and increased expectoration (p<0.05). Analysis using a univariate approach indicated significant correlations between the highest body temperature during fever,the cycle threshold at the initial detection,and expectoration with pneumonia (p<0.05). Univariate logistic regression showed that the initial cycle threshold was significantly associated with pertussis-associated pneumonia (OR=1.483, p<0.001); multivariate logistic regression further confirmed it as an independent risk factor (OR=0.675, 95 % CI: 0.542–0.839, p<0.001). The group affected by pneumonia administered higher usage of erythromycin/cephalosporins (p<0.05). Both univariate and multivariate logistic regression analyses revealed a substantial relationship between the initial detection cycle threshold and pneumonia (p<0.05). 54.6 % patients had co-infections. In our pediatric population, the most commonly identified pathogens were human rhinovirus, mycoplasma pneumoniae and respiratory syncytial virus.

**Conclusions:**

The cycle threshold values were a risk factor for pneumonia in children with B. Pertussis infection,a lower initial Ct value (indicating higher bacterial load) is a significant risk factor for developing pneumonia in children with pertussis. There was higher usage of erythromycin/cephalosporins in the pneumonia group. While B. Pertussis has a high coinfection rate in childhood infections, mainly with HRV,MP and RSV, which also highlighed the importance of comprehensive pathogen detection. Clinical doctors should fully consider the above situation in children with B. Pertussis infection to diagnose and treat correctly.

## Highlight box

### Key findings

The cycle threshold values was risk factor for pneumonia in children with B. Pertussis infection accompanied by the highest body temperature during fever and expectoration.

While B. Pertussis has a high coinfection rate in childhood infections, mainly with HRV, MP, and RSV.

### What is known and what is new?

Bordetella pertussis infection can cause pneumonia in children, but research on other related risk factors and coinfections were still lacking.

This study emphasizes the risk factor for B. pertussis-associated pneumonia is the cycle threshold value.The most commonly coinfection pathogens were human rhinovirus, mycoplasma pneumoniae and respiratory syncytial virus.

### What is the implication, and what should change now?

Clinical doctors should pay attention to the cycle threshold value of children with B. Pertussis infection, especially when there is excessive sputum and high fever, and be alert to the occurrence of pneumonia. Moreover, B. Pertussis infection can be coinfections, mainlyhuman rhinovirus, mycoplasma pneumoniae and respiratory syncytial virus.

Future research should explore the mechanism of B. Pertussis infection and develop more effective B. Pertussis vaccines.

## Introduction

Pertussis, an acute respiratory infectious disease triggered by *Bordetella pertussis* (B.pertussis), spreads through respiratory aerosols. It presents significant morbidity and mortality risks among children [1], 2]. Over the past 40 years, whole-cell pertussis vaccines have proven highly effective, averting approximately 760,000 deathsglobally each year. Despite this,the burden of pertussis remains considerable, with around 50 million cases and 300,000 fatalities every year, predominantly among infants. Symptoms generally appear 7–10 days after infection, beginning with a slight fever, a runny nose, and a cough, which frequently develops into a severe, hacking cough accompanied by a whooping sound. Pneumonia is a common complication associated with the disease. Antibiotics are used to treat the infection.

*Bordetella pertussis*, a gram-negative bacterium identified initially in 1906, infects humans through the upper respiratory system. This infection results in inflammation of the respiratory system, possible harmful effects,and a decrease in immune function. Youngsters infected with *Bordetella pertussis* face a higher risk of opportunistic infections and are more susceptible to various other viruses, bacteria,and pathogens [3].

The gradual introduction of whole-cell pertussis vaccines (WCV) worldwide in the mid-1940s was followed by a dramatic decrease in illness and death from pertussis [4]. In China, pertussis immunization with WCV was introduced in the early 1960s. Since 1978, when China incorporated the whole-cell pertussis vaccine (WCV) along with diphtheria and tetanus toxoids into its expanded immunization scheme, this vaccine has demonstrated a significant impact on lowering morbidity and mortality rates in infants and young children [5], 6]. Nevertheless, the safety and potential side effects associated with the WCV have attracted substantial global public concern. In 2013, China began using the acellular pertussis vaccine (ACV), produced through co-purification techniques. This vaccine primarily consists of pertussis toxin and filamentous hemagglutinin, along with several additional antigens that cannot be entirely eliminated.

Although vaccination rates have reached high levels, the occurrence of pertussis has been increasing in many nations in recent decades, making it an important global public health issue. In 2018, there were over 151,000 reported cases of pertussis worldwide. By 2022, this number decreased to more than 62,500 cases globally, with nearly 39,000 instances documented in the Western Pacific Region. For developing nations, an estimate suggests that the typical case fatality rate (CFR) for pertussis is around 4 % in infants under the age of one and about 1 % for children aged one to four years. From 2007 to 2022, Jiangsu Province in China reported the highest number of cases among children under one year old [7].

This research aims to investigate the clinical features and identify factors related to pneumonia in children with B. Pertussis infection. We present this article in accordance with the STROBE checklist for reporting.

## Materials and methods

### Study population

A total of 75 cases met the inclusion criteria and were enrolled in the study. These patients were admitted to the Women’s and Children’s Hospital affiliated with NingboUniversity in Ningbo, Zhejiang, China, from January 2024 to April 2024. The diagnostic criteria for *Bordetella pertussis* adhered to the “Consensus of Experts on Laboratory Detection and Quality Management of Pertussis in China (2024 Edition),” established by the Branch of Clinical Diagnosis and Laboratory Medicine of the China Maternal and Child Health Association. The criteria included: (1) a paroxysmal spastic cough; (2) vomiting that follows coughing; (3) unexplained paroxysmal cyanosis or asphyxia in newborns or infants, often without a typical spastic cough; (4) a persistent cough lasting 14 days or more, with other causes excluded;and the presence of at least one of these four criteria, along with (5) detection of Bordetella. pertussis nucleic acid via PCR [8]. The exclusion criteria comprised: (1) age over 18 years; (2) severe dysfunction of heart, liver,or kidney organs; (3) congenital abnormalities in airway development;and (4) coexisting immune system disorders.

### Criteria for diagnosing pneumonia

The study participants were divided into two categories:pertussis patients with pneumonia (pneumonia group) and those without pneumonia (non-pneumonia group). The criteria used to diagnose pneumonia followed the “Guidelines for the Management of Community-Acquired Pneumonia in Children” (revised edition, 2013), which were set forth by the Respiratory Group within the Pediatrics Branch of the Chinese Medical Association [9]. The observed symptoms included: (1) clinical manifestations such as fever, cough,dyspnea, tachypnea,and chest wall retraction; (2) respiratory signs identifiable through lung auscultation, including moist rales and tubular breath sounds; and (3) Imaging results throughout the treatment process, including chest X-rays or CT scans,may show abnormal lesions that are either unilateral or bilateral, mainly observed as central shadows in the lung areas, irregular shadows,and alterations in the bilateral interstitial regions. Additionally, instances of segmental consolidation or pleural effusion may also be present.

### Data gathering

The medical records of 75 pediatric patients were examined to collect information regarding age, gender, clinical signs, and results of laboratory tests. The assessment criteria encompassed gender, age, the peak temperature noted, status of influenza vaccination, presence of cough, sore throat, nasal blockage, nasal discharge, sputum production, wheezing, cyanosis associated with cough, difficulty in breathing, vomiting,and diarrhea. Throat and blood samples were collected within 24 h following admission. The laboratory analyses involved detecting B. Pertussis DNA, along with testing for thirteen common viruses: respiratory syncytial virus (RSV), human rhinovirus (HRV), human bocavirus (hBoV), influenza virus A (IFV-A), influenza A virus subtype H1N1 (IFV-A H1N1), influenza A virus subtype H3N2 (IFV-A H3N2), influenza virus B (IFV-B), human parainfluenza virus (HPIV), human metapneumovirus (HMPV), adenovirus (ADV), mycoplasma pneumonia (MP), *Chlamydophila pneumoniae* (CPn), and human coronavirus (hCoV). Additionally, the proportions of peripheral white blood cells (WBC) and leukomonocytes (LN), as well as high-sensitivity C-reactive protein (hsCRP) levels, were analyzed.

### Laboratory-based test methods

#### B. Pertussis-DNA detection

Samples from throat swabs were acquired within 24 h of admission to the hospital and examined with the SLAN-96S real-time fluorescence quantitative PCR device by Shanghai Hongshi Medical Technology Co.,Ltd. The analysis kit utilized for this procedure was obtained from Shenzhen Yilifang Technology,and the protocol was strictly adhered to as per the given guidelines.

#### Detection of thirteen common viruses

Throat swab samples were taken within 24 h after the patient’s admission to the hospital. Detection of thirteen respiratory tract pathogens was performed using the Thirteen Respiratory Tract Pathogens Detection Kit developed by Ningbo Haisch Biotechnology Co.,Ltd. This analysis employed PCR capillary electrophoresis fragment analysis as the detection method.

#### Blood routine and high-sensitivity C-reactive protein detection

A volume of 2 mL of peripheral blood was collected in a procoagulant tube, and the samples were analyzed using the Mindray BC-5390 Series Fully Automatic Hematology Analyzer.

#### Statistical analysis

Statistical evaluations were performed utilizing SPSS version 19.0 software. Continuous variables that adhered to a normal distribution are represented as the mean ± standard deviation.Independent-sample t-tests were applied to compare the groups. For data that did not meet normal distribution criteria, results are presented as M(P25, P75), and the Mann-Whitney U test was used to evaluate the differences between the two groups. The analysis of categorical data was carried out using the χ^2^ test. Comparisons were made between the pneumonia group and the non-pneumonia group, including significant indicators in the univariate analysis. Variables with p<0.05 in univariate analysis were included, and collinear factors were excluded via variance inflation factor (VIF) analysis (VIF>10 defined as collinearity). Factors exhibiting collinearity with others were omitted, while the other variables were retained for further examination using the multiple logistic regression model. Although maximum fever and expectoration were significant in univariate group comparisons (p<0.05, Table 1), they were not included in the multivariate regression model because they were non-significant in univariate logistic regression (p>0.05, Table 4). All p-values were calculated using a two-tailed test, with a significance threshold set at a p-value of less than 0.05.

**Table 1:** The general data and clinical characteristics of 75 pertussis patients.

	No. (%)patients			
Evaluation indicator	Total patients (n=75)	Pneumonia group (n=29)	Non-pneumonia group (n=46)	p-Value
Sex				
Boys	39 (52)	14	25	0.61
Girls	36 (48)	15	21	
Age [year, M(P25, P75)]	6.0 (3.583, 8.0)	6 (0.44, 8.0)	6 (5.25, 8.0)	0.31
Maximum body temperature [C, M(P25, P75)]	36.50 (36.50, 36.8)	36.50 (36.5, 37.0)	36.50 (36.50, 36.62)	0.03
The cycle threshold of the first detection [value, M(P25, P75)]	21.58 (18.73, 24.70)	18.49 (16.88, 21.19)	23.08 (20.29, 27.56)	0.01
Fever				
Yes	8 (10.7)	5	3	0.25
No	67 (89.3)	24	43	
Pertussis vaccine (case)				
Yes	58 (77.3)	19	39	0.05
No	17 (22.7)	10	7	
Cough (case)				
Yes	73 (97.3)	28	46	0.39
No	2 (2.7)	1	0	
Throat pain (case)				
Yes	5 (6.7)	3	2	0.37
No	70 (93.3)	26	44	
Nasal congestion				
Yes	2 (2.7)	0	2	0.52
No	73 (97.3)	29	44	
Nasal mucus				
Yes	40 (53.3)	3	37	0.56
No	35 (46.7)	26	9	
Expectoration				
Yes	57 (76.0)	17	40	0.005
No	18 (24.0)	12	6	
Wheezing				
Yes	3 (4.0)	3	0	0.054
No	72 (96.0)	26	46	
Cyanosis during coughing				
Yes	2 (2.7)	2	0	0.15
No	73 (97.3)	27	46	
Shortness of breath				
Yes	2 (2.7)	2	0	0.15
No	73 (97.3)	27	46	
Vomit				
Yes	3 (4.0)	2	1	0.56
No	72 (96.0)	27	45	

### Ethical statement

This study protocol was reviewed and approved by The Affiliated Women and Children’s Hospital of Ningbo University Ethics Committee.

## Results

### Patient information

**Figure 1:**
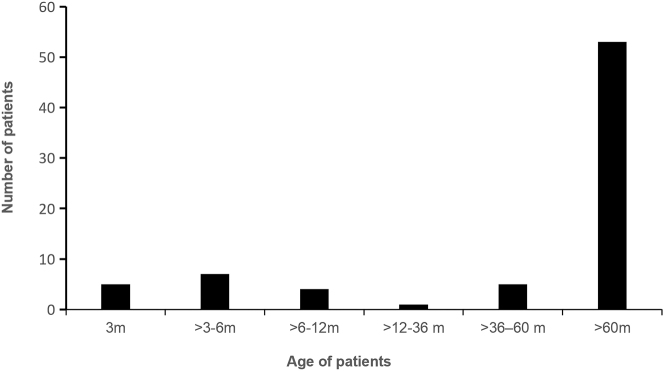
Distribution of ages among patients diagnosed with pertussis infection.

A total of 75 individuals participated in the study, comprising 39 males (52 %)and 36 females (48 %). The average age of the patients was 5.70 ± 3.55 years, spanning from 2.65 months to 18 years. Figure 1 illustrates the age distribution, revealing that 53 patients (70.7 %) were older than 60 months. Among the participants, 58 individuals (77.30 %) had completed the full course of the pertussis vaccine, while 17 individuals (22.70 %) had not. Based on the pneumonia diagnosis, patients were categorized into two groups: 29 in the pneumonia group and 46 in the non-pneumonia group, as detailed in Table 1. All children enrolled in the study exhibited varying degrees of paroxysmal cough. A small number experienced respiratory symptoms, including throat pain, nasal congestion, nasal mucus, expectoration,wheezing, cyanosis during coughing,and shortness of breath. Additionally,a small number reported gastrointestinal symptoms such as vomiting. None exhibited symptoms of diarrhea or febrile convulsions.

### Pairwise comparison of each evaluation index

A statistically significant difference (p<0.005) was observed between the pneumonia group and the group without pneumonia regarding the highest recorded body temperature, initial cycle threshold values,and expectoration rates. There was borderline significance between the completion of vaccination and the occurrence of pneumonia (p=0.05). In contrast, no statistical significance was found when comparing other indicators between these two groups (p>0.05; see Tables 1 and 2). Among the 75 cases, 38 (50.7 %) were treated with azithromycin, 21 (28.0 %) with cephalosporins, and 16 (21.7 %) witherythromycin.A significant difference in the use of erythromycin compared tocephalosporins was observed between the pneumonia group and the group without pneumonia (p<0.05), as depicted in Table 2.

**Table 2:** A comparison of drug results and laboratory tests between the two groups.

Factors	Total patients (n=75)	Pneumonia group (n=29)	Non-pneumonia group (n=46)	p-Value
Laboratory index				
WBC, ×109/L	9.4 (7.2, 12.7)	8.9 (6.7, 15.25)	9.45 (7.35, 12.20)	0.89
LY, %	38.93 ± 18.81	42.17 ± 20.04	36.89 ± 17.90	0.24
NEU, %	51.79 ± 19.95	47.55 ± 22.40	54.46 ± 17.98	0.15
hsCRP, mg/L	1.1 (0.3, 2.7)	0.5 (0.1, 1.7)	1.4 (0.5, 3.625)	0.6
Drug				
Azithromycin				
Yes	38 (50.7)	14	24	0.74
No	37 (49.3)	15	22	
Erythromycin				
Yes	21 (28.0)	14	7	0.001
No	54 (72.0)	15	39	
Cephalosporin				
Yes	16 (21.3)	12	4	0.002
No	59 (78.7)	17	42	

WBC, white blood cell; LY, lymphocyte; Neu, neutrophil; hsCRP, high-sensitivity C-reactive protein.

### Correlation analysis of the CT value of the first detection with maximum body temperature and expectoration

Spearman’s rank correlation was applied to analyze its associations with expectoration and body temperature. There was a statistically significant positive correlation between the CT value of the first detection and expectoration (ρ=0.49, 95 % CI: 0.32–0.63, p<0.001). However, there was no correlation between the CT value of the first detection and the maximum body temperature, as depicted in Table 3.

**Table 3:** Spearman’s rank correlation analysis of the CT value of the first detection with maximum body temperature and expectoration (n=75).

Indicator	ρ	p-Value	95 % CI
Maximum body temperature, °C	0.10	0.38	(−0.12, 0.31)
Expectoration	0.49	0.000	(0.32, 0.63)

CI, confdence interval.

### Regression analysis was used to evaluate the correlation between each index and pneumonia

The univariate analysis revealed a significant correlation between the cycle threshold of the initial detection and pneumonia (p<0.05). Additionally, the multivariate logistic regression analysis validated a notable correlation between the cycle threshold at first detection and pneumonia (p<0.05), as illustrated in Table 4.

**Table 4:** Analysis of risk factors for pneumonia complicated by *Bordetella pertussis* using univariate and multivariate logistic regression techniques.

Indicator	Univariate logistic analysis			Multivariate logistic regression analysis			VIF
	OR	95 % CI	p-Value	OR	95 % CI	p-Value	
Maximum body temperature, °C	0.757	(0.314, 1.824)	>0.05	1.322	(0.548, 1.326)	0.53	1.029
The cycle threshold of the first detection	1.483	(1.192, 1.844)	<0.001	0.675	(0.542, 0.839)	<0.001	1.029
Expectoration	0.395	(0.101, 1.544)	>0.05	0.395	(0.301, 1.544)	0.18	1.000

OR, odds ratio; CI, confdence interval.

### Co-infections status related to pertussis infection

**Figure 2:**
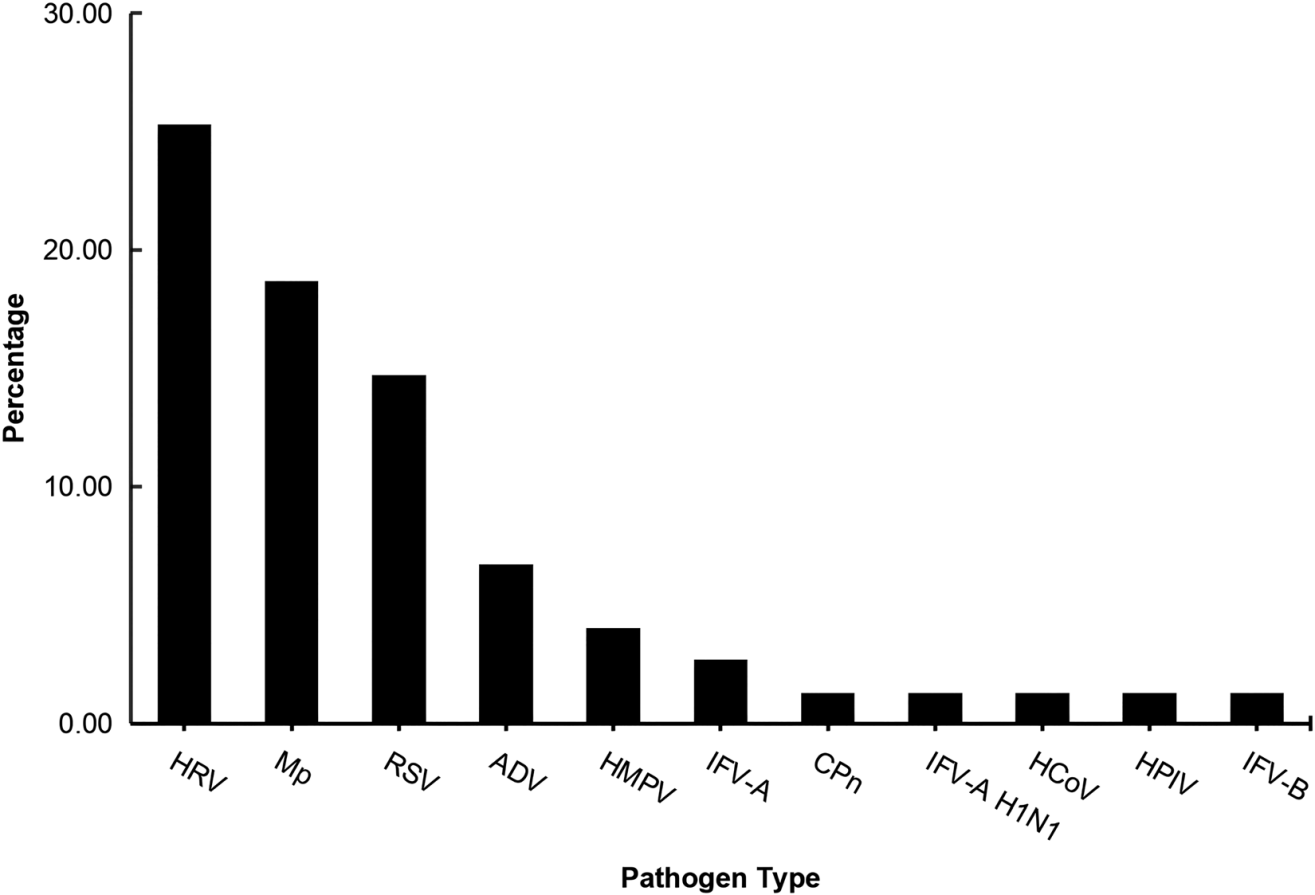
Illustrates the distribution of coinfections related to pertussis.

Out of 75 children who were diagnosed with pertussis, 41 (54.6 %) exhibited coinfections with several other respiratory pathogens. This included 19 (25.3 %) cases of human rhinovirus (HRV), 14 cases (18.7 %) with *Mycoplasma pneumoniae* (MP), 11 cases (14.7 %) with respiratory syncytial virus (RSV), 5 cases (6.7 %) with adenovirus (ADV), 3 cases (4.0 %) with human metapneumovirus (HMPV), 2 cases (2.7 %) with influenza virus A (IFV-A), including one case of the IFV-A H1N1 subtype, 1 cases (1.3 %) with influenza virus B(IFV-B), and coinfections with human parainfluenza virus (HPIV), *Chlamydophila pneumoniae* (CPn), and human coronavirus (hCoV). No cases were found with human bocavirus (hBoV) or influenza virus A subtype H3N2(IFV-A H3N2). The distribution of two and three coinfections is illustrated in Table 5 and Figure 2.

**Table 5:** Pathogen coinfections correlated with pertussis infection (n=75).

Pathogens			Number
One-coinfection			
HADV			1
HRV			10
HMPV			1
IFV-B			1
MP			4
HRSV			9
Two-coinfections			
IFV-A	MP		1
IFV-A	HADV		1
HADV	MP		1
HRV	MP		6
HRV	HMPV		1
HRV	HPIV		1
CPn	HRSV		1
hCoV	HRSV		1
Three-coinfections			
HADV	HMPV	MP	1
HADV	HRV	MP	1

In this context, HRV refers to human rhinovirus, MP refers to *Mycoplasma pneumoniae*, RSV refers to respiratory syncytial virus, and ADV signifies adenovirus. HMPV refers to human metapneumovirus, IFV-A indicates influenza virus A, IFV-A H1N1 refers toinfluenza A virus subtype H1N1, IFV-B indicates influenza virus B, HPIV indicates human parainfluenza virus, CPn indicates *Chlamydophila pneumoniae*, hCoV indicates human coronavirus, IFV-A H3N2 indicates influenza A virus subtype H3N2, and hBoV indicates human bocavirus.

## Discussion

This study examined the clinical features of 75 individuals who were diagnosed with pertussis via PCR testing in Ningbo, a coastal city located in the southeastern region of China. Of these individuals, 39 (52 %) were male, while 36 (48 %) were female. The study identified pertussis cases spanning a broad age spectrum, ranging from 2.65 months to 17 years. Notably, the highest positivity rate of 70.7 % was found in children older than 60 months. However, prior research has indicated that the distribution of pertussis cases by age differs across various global regions. During an outbreak in the United States, according to experts’ analysis, the prevalences of pertussis were particularly significant among infants younger than 2–3 months in all countries for which data was available. Additionally,a markedly high incidence was noted in vaccinated children aged between 14 and 16 years. Surveillance data from 204 countries and territories gathered from 1990 to 2019 showed that the highest incidece and mortality rates of pertussis occurred in infants under 1 year old,with the 1–4 year age group following as the second most affected in terms of disease burden [10]. Meanwhile,a remarkably high incidence was also observed in children aged between 14 and 16 years who have had vaccinations against pertussis [11]. A surveillance studyconducted across 204 countries and territories from 1990 to 2019 revealed that the highest incidence and mortality rates of pertussis occur in the age group under 1 year old.Additionally,the age group of 1–4 years old ranks second in terms of disease burden [12].

In this research, individuals diagnosed with pertussis were categorized into two groups:those with pneumonia and those without. The analysis showed no statistically significant differences in age or gender between the two groups. Additionally, symptoms such as coughing, sore throat,and other indicators of respiratory infection did not display significant variations across the groups. This finding contrasts with some studies on general respiratory diseases, where these symptoms are often deemed important indicators of disease severity and classification. However, the pneumonia group in our study exhibited higher maximum fever and increased expectoration (p<0.05). For instance, research on lower respiratory tract infections has demonstrated that elevated body temperature and sputum production was strongly linked to occurrence and degree of pulmonary inflammation. One study specifically noted that elevated fever in pertussis cases frequently correlates with bacterial coinfections [13]. From a pathogenesis perspective, after *Bordetella pertussis* infects the human body, it multiplies extensively within the respiratory tract and releases various toxins. These toxins damage the respiratory mucosa, resulting in mucosal congestion, edema,and increased mucus secretion [14]. Coughing up sputum may serve as an early warning sign of pneumonia induced by pertussis. The cycle threshold (Ct) value for initial detection was significantly lower in the pneumonia group compared to the non-pneumonia group. Expectoration is correlated with the Ct value for initial detection. The analysis of nasopharyngeal samples through polymerase chain reaction (PCR)is widely acknowledged as the most dependable approach for identifying *Bordetella pertussis* [15]. Another study proposed that the cycle thresholdvalues from real-time PCR for pertussis could serve as a indicator of severe disease in infants and young children, suggesting that an elevated bacterial load could correlate with more severe cases. This correlation arises from the fact that a substantial bacterial load can result in enhanced toxin production and other associated factors [16]. In infections caused by *Bordetella pertussis*, symptoms typically manifest following the peak of the infection.

In our study, we identified additional factors associated with pertussis pneumonia. There were no statistically significant variations in the white blood cell (WBC) count, the percentages of lymphocytes and neutrophils, or the levels of C-reactive protein (CRP) when comparing between pneumonia group and non-pneumonia group. This lack of significance may be attributed to the timing of our testing, which resulted in variations in quantitative evaluation. Some patients had already commenced empirical antibiotic treatment prior to the measurement of these indicators, potentially affecting the WBC count and other parameters during testing. Vaccination completion showed borderline significance with the occurrence of pneumonia (p=0.05), suggesting a potential protective effect of pertussis vaccination. Notably, patients in the pneumonia cohort demonstrated significantly higher utilization rates of erythromycin and cephalosporins compared to the non-pneumonia group (p<0.05). Patients in the pneumonia group received erythromycin and cephalosporins more frequently, likely reflecting clinician response to more severe disease or concern for bacterial co-infection. This observation is consistent with current therapeutic paradigms: erythromycin, a key macrolide antibiotic, directly inhibits the proliferation of *Bordetella pertussis* by obstructing bacterial protein synthesis [17]. Furthermore, the frequent adjunctive use of cephalosporins in pneumonia cases likely reflects clinicians’ proactive management of suspected secondary bacterial coinfections, a situation that is strongly linked to pulmonary parenchymal involvement. Such empirical combination therapy may targetpathogens like *Streptococcus pneumoniae* and *Haemophilus influenzae*, which frequently complicate airway damage associated with pertussis [18].

The identification of coinfection has been documented in earlier research, which demonstrates variability across different regions. Scutari et al. indicated that coinfection was present in 76.7 % of cases within their pediatric cohort, predominantly associated with HRV, HMPV, and HPIV [19]. In Italy, Nicolai et al.observed a viral coinfection rate of 47.2 % in infants hospitalized due to pertussis, with frequently identified pathogens being HRV, RSV, and hCoV [20]. Jiang et al.concluded that viruses coinfection was a modifiable risk factor for severe pneumonia in B.pertussis-infected children [21]. A recent investigation carried out in Huan Province, China,found that 439 instances (58.1 %) displayed mixed infections, and RSV was recognized as the predominant pathogen, accounting for 38.7 % [22]. It is important to note that their investigation was limited to testing only seven prevalent viruses: RSV, IVA, IVB, PIV(I, II, III), and ADV. Importantly,key co-pathogens identified in our research, such as HRV, MP, and HMPV, were not assessed in their analysis. The occurrence rate of coinfections in our study was noted to be 54.6 %. Notably,in southeastern China, HRV was identified as the pathogen most frequently co-detected at 25.3 %, with MP at 18.7 % and RSV at 14.7 % following. These findings suggest that variations in environmental factors, hygiene practices, vaccination prevalence,and the types of vaccines available across different regions contribute to a diverse array of pathogens that co-infect with pertussis. However, the study faced several limitations. Firstly, we only collected pertussis cases from a single hospital in Ningbo. Future research should involve collecting cases from multiple hospitals across different regions. Secondly, there is a possibility that certain patients had coinfections with other pathogens that were not detected. A major limitation is the 4-month study period (January–April 2024), as pertussis and other respiratory viruses have distinct seasonal variations. This short duration may significantly skew the co-infection results (e.g., underrepresenting pathogens prevalent in other seasons) and limits the generalizability of the findings to broader populations. The small sample size, especially of the pneumonia group (n=29), should be explicitly stated as a key limitation that affects the statistical power and the robustness of the multivariate regression model. Data sources will be expanded in future research.

We found a lower initial Ct value (indicating higher bacterial load) is a significant risk factor for developing pneumonia in children with pertussis, and combination therapy of erythromycin/cephalosporins may be administered in the pneumonia group. Additionally, *B. pertussis* infection in children is associated with a high co-infection rate (54.6 %), mainly involving human rhinovirus (HRV), *Mycoplasma pneumoniae* (MP), and respiratory syncytial virus (RSV), highlighting the importance of comprehensive pathogen detection for accurate diagnosis and treatment.
